# Preliminary Support for a Generalized Arousal Model of Political Conservatism

**DOI:** 10.1371/journal.pone.0083333

**Published:** 2013-12-20

**Authors:** Shona M. Tritt, Michael Inzlicht, Jordan B. Peterson

**Affiliations:** 1 Department of Psychology, University of Toronto, Toronto, Canada; 2 Department of Psychology, University of Toronto Scarborough, Toronto, Canada; The University of New South Wales, Australia

## Abstract

It is widely held that negative emotions such as threat, anxiety, and disgust represent the core psychological factors that enhance conservative political beliefs. We put forward an alternative hypothesis: that conservatism is fundamentally motivated by arousal, and that, in this context, the effect of negative emotion is due to engaging intensely arousing states. Here we show that study participants agreed more with right but not left-wing political speeches after being exposed to positive as well as negative emotion-inducing film-clips. No such effect emerged for neutral-content videos. A follow-up study replicated and extended this effect. These results are consistent with the idea that emotional arousal, in general, and not negative valence, specifically, may underlie political conservatism.

## Introduction

The vast majority of psychological theories of political orientation are predicated on the assumption that negative emotional states enhance conservative belief (e.g., [1]–[4]). In support of this notion, exposure to fear-inducing and disgusting stimuli motivate conservative versus liberal shifts in political beliefs (See [4] for review).

The negatively-valenced emotional states that have previously been linked to conservativism, however, are highly arousing as well as unpleasant. We have recently argued that this confound of valence and arousal may have led to the mistaken conclusion that negative valence *per se* is associated with conservative political beliefs rather than arousal more generally [5]. Little is known, for instance, about the impact of positively-valenced arousal upon political orientation.

In this context, we explored the possibility that emotionally arousing positive as well as negative stimuli would lead to enhanced agreement with conservative political content. Such a finding might suggest that arousal, rather than negative valence, underlies the psychological motivations to endorse conservative political belief.

### Arousal and valence confounded in political psychology research

Experimental studies have suggested that aversive emotional manipulations lead to conservative shifts in political beliefs. For instance, experimentally-induced as well as real-world threat leads to shifts toward conservative beliefs (e.g., [6]–[9]). Experimental induction of disgust has also been found to lead to conservative shifts [10]–[13]. These findings have been interpreted as evidence that conservative political orientation is motivated by negative emotional states [4]. However, the experimental studies that have manipulated emotion and assessed its impact upon political ideology have neglected to assess the impact of non-negative forms of arousal.

It is premature to conclude that negative valence causes conservative shifts when the impact of positively-valenced arousing stimuli has not been assessed. To our knowledge, only one experimental study has included positively valenced stimuli, in particular, happy faces [14]. Yet because happy faces have elsewhere been found to be less motivationally salient/arousing than unhappy/angry faces [15], it remains unclear whether valence or arousal underlie such findings.

Arousal and valence have often been confounded. Psychologists assumed for decades that humans have a negativity bias, responding more intensely to negative than to positive and/or neutral information (e.g., [16]–[18]). However, some of the support for such a bias seems to have come from the use of positive stimuli that are low in arousal—i.e., calming stimuli like pictures of pleasant scenery, instead of stimuli that are high in arousal such as erotica. Recently, psychophysiological studies have suggested that individuals exhibit biased processing of highly arousing compared to neutral stimuli—regardless of whether those stimuli are positive or negative (e.g., [19]). In this context, it seems that humans respond most intensely to stimuli that are motivationally salient, rather than manifesting a negativity bias *per se*.

Political psychology research seems to have, in this vein, confounded valence and arousal, which may have led to the false conclusion that negative valence *per se* is associated with conservative political beliefs. For example, some studies have examined the way that conservatives process highly arousing, negative stimuli compared to less-arousing, positively valenced information, confounding the effects of arousal and valence [20]–[22]. Among these, Carraro and colleagues [20] assessed attentional bias to calming positive stimuli (e.g., words such as peace and serenity, pictures of flowers) compared to more arousing negative stimuli (e.g., words such as vomit and terror, pictures such as animals barring their teeth). This study found a relationship between conservative ideology and increased reactivity to negative rather than positive stimuli. However, it remains unclear whether these effects are due to an increased sensitivity to negative stimuli, as argued by the authors, or an increased sensitivity to arousing stimuli. Indeed, we believe that there is reason to suspect that arousal rather than valence may underlie such conservative ideological tendencies.

Consistent with this notion, we recently found that conservatives, compared to liberals, exhibited enhanced electrophysiological sensitivity to motivationally salient positive stimuli [23]. This suggests that conservatives exhibit attentional bias to salient stimuli not specific to negative valence. In an additional electrophysiological study, we used multi-level modeling to assess whether conservatives exhibit physiological sensitivity to arousal or valence, controlling for the relative impact of each [24]. In this highly powered study, we found a significant interaction between political orientation and arousal but not valence in predicting physiological sensitivity. Taken together, these two studies support the role of arousal rather than valence as the affective component of conservative political orientation [23]–[24]. Nevertheless, while these studies shed light upon trait-level individual differences in arousal sensitivity, the causality of the relationship between conservative orientation and sensitivity to arousal remains unknown.

### Political conservatism: Motivated by arousal, not specifically threat and uncertainty

We suspect that arousal may induce conservative shifts in political orientation. Why? At least two possibilities appear to exist: 1) arousal motivates individuals to endorse value systems that promote societal structures that minimize the potential for intense arousal, and 2) arousal interferes with cognitive ability, which causes a preference for intuitive ideas, of which conservative ideas are one type.

The first possibility is predicated on the assumption that individuals, weary of existence in an overly aroused state, endorse political conservatism in an attempt to regulate emotion or motivation-inducing environmental contexts. Although the activation of primitive arousal systems is frequently biologically beneficial, driving organisms to eat, drink, procreate, and to avoid danger, intense arousal is also commonly experienced as aversive [25]–[26]. Arousal may interfere with controlled thought processes and prompt individuals to action motivated by potentially disruptive, even dangerous, short-term impulse, rather than under the guidance of reason, agreed upon rules, and duties. Even moderate levels of positive emotion, subjectively desirable as they might be, can result in increases in future discounting, with a disregard for medium to long term security [27], while extreme levels are clearly associated with impulsivity and mania [28]. Furthermore, and somewhat counter-intuitively, positive emotion can also interfere with the maintenance of social order, as attested to by the frequency and severity of celebratory riots [29]. It seems then that although suppressing hedonistic instincts is draining (see [30]), it is often less dangerous—at the individual and the societal level–than engaging in such impulses.

We believe that the motivation to avoid intense arousal—at least to some degree—is common among humans. Nonetheless, there are individual differences in the extent to which people are hedonistic versus inhibited. Recent personality studies provide support for the idea that conservatives tend to be particularly intolerant of emotional arousal. Leone and Chirumbolo [31], for example, suggested that conservatives generally avoid emotional experience, and are less inclined to indulge feelings of any type. Moreover, conservatives are more likely to have been reared in authoritarian households, where the presumption is that behavior should be governed by external rules and sanctioned authority figures rather than motivated by personal impulses or feelings [32]. Converging evidence, therefore, suggests that conservatives are inclined to avoid intense motivational and emotional arousal. Individuals may adopt a conservative lifestyle and accept conservative beliefs in an attempt to avoid environmentally–and psychologically–caused arousal. Conservatives generally support the current status-quo, minimizing the need for change. More specifically, they also advocate for stricter immigration policies and provide little support for the existence of alternative lifestyles. This logically helps minimize exposure to novel value systems. By offering a common set of externally prescribed and fixed values, to which all are optimally subject, political conservatism may provide the individual with a means of regulating the social environment to limit exposure to emotionally and motivationally arousing situations.

A second possible mechanism whereby intense arousal may lead individuals to prefer conservative ideas is through simple interference with cognitive ability. Emotional arousal inhibits performance on cerebrally-taxing cognitive tasks. Positive (see [33] for review; [34]–[36]) as well as negative [37]–[40] emotional induction has been found to lead individuals to rely on *gut-level* rather than controlled cognitive processes. The neurobiological mechanism through which this occurs has been tentatively mapped. Emotional distractions evoke activity in brain regions such as the amygdala and ventrolateral prefrontal cortex, while deactivating activity in the working memory regions such as the dorsolateral prefrontal cortex, thereby hindering performance on working memory tasks [41], which are central to controlled cognitive function.

We suspect, as have others [42], that conservative political belief is linked to fast and efficient information processing requiring comparatively little effort, time, or awareness. In support of this idea, experimentally-induced gut-level rather than controlled cerebral processing has in fact been found to enhance conservatism. Eidelman and colleagues [42] demonstrated, for instance, that conservative political beliefs were augmented whenever effortful thought-processing was disrupted–by factors as diverse as alcohol intoxication, cognitive load, and time pressure. Moreover, cognitive ability is inversely correlated with conservative political beliefs (e.g., [43]). It seems conceivable, then, that emotional and motivational arousal interferes with effortful cognitive processing, and this subsequently enhances the probability of adopting conservative beliefs.

In sum, conservative ideology may be attractive to individuals who are in a state of arousal because it minimizes potential for further arousal *and* because it is intuitive. In this experimental study, we assessed whether emotional arousal—both positive and negative—would lead to conservative shifts in political orientation.

## Research Overview

In a series of two studies, we assessed the validity of the arousal versus the negative valence hypotheses of the psychological motivations underlying political conservatism. We accomplished this by investigating whether amusement, a non-threatening, non-uncertainty-related form of arousal, would cause enhanced endorsement of conservative political belief.

### Study 1

Previous studies have found that experimentally-induced as well as real-world threats (e.g., [6]–[9] and disgust (e.g., [10]–[13]) lead to shifts toward conservative beliefs. Such findings have been interpreted as evidence that threat and disgust, specifically, enhance the attractiveness of conservative ideology. Study 1 investigates an alternative possibility, that a broad range of arousal motivates individuals to endorse more conservative political beliefs. We experimentally induced several types of emotional arousal states to see if this would also lead to conservative shifts in beliefs. In particular, we exposed participants to one of several film clips with contents designed to produce a variety of states of arousal, or one of several neutral film clips, and asked them to indicate their subsequent agreement with right or left-wing political speeches. Two competing hypotheses were tested: 1) if the negative valence hypothesis was correct, then scary and disgusting film-clips, compared with positive and neutral film-clips, should increase agreement with right-wing speeches; 2) if political conservatism is motivated by arousal, more broadly, then a range of emotionally-arousing, compared with neutral film-clips, should lead to increased agreement with right-wing political speeches.

Emotionally arousing compared to neutral film clips were additionally expected to decrease agreement with left-wing speeches. This is because left and right political views have traditionally been thought to form opposed ends of a bipolar spectrum (see [44] for a review), which means that enhanced agreement with conservative ideas should be correlated with decreased agreement with liberal ideas. However, this hypothesis was largely exploratory because a number of authors have argued that political orientation is not best represented by a single dimension ranging from liberalism to conservatism but rather that the left and right represent two independent unipolar dimensions (e.g., [45]–[46]). In support of this notion, exploratory and confirmatory factor analyses have suggested that evaluation of left and right wing ideas load onto different latent variables that are at least somewhat independent of each other (e.g., [46]).

### Method

#### Participants

578 American participants were recruited from Amazon's Mechanical Turk (MT), an online platform for enlisting workers, and each was compensated $1.20 for participation in the study. Participants recruited from MT are more representative of the United States population than traditional subject pools (i.e., undergraduate samples) with regards to gender, race, age, and education [47]–[50]. Due to attrition, a final sample of 442 completed the study in its entirety (240 males with a mean age of 31.91years; *SD* = 23.37; range 18–78). In lengthy studies run on MT, it is normal for such a large number of participants not to complete the study.

#### Political orientation

Baseline political orientation was assessed with the question: “Please rate yourself on a scale ranging from 1 (liberal) to 7 (conservative).” This method of assessing political belief with a one-item dimensional scale has been used previously (e.g., [44], [51]). The sample mean was 3.50 (SD = 1.84).

#### Procedure

The study employed a between-groups design. Participants were randomly assigned to watch either an emotion-inducing or a neutral film clip and then to read either left-wing or right-wing content speeches. Participants rated their level of agreement with political speeches using a 9-point Likert scale ranging from 1 (I do not agree at all) to 9 (I completely agree).

The research protocol was approved by the University of Toronto ethics board. All participants gave their consent before beginning the online survey.

#### Film clips

Participants were randomly assigned to view a film clip that was frightening, amusing, disgusting, or neutral, in order to induce emotional arousal or neutral affect. A between-groups design was used to eliminate carry-over effects of different types of emotional arousal. To ensure that the effects were not stimulus specific, we used three different film clips of each type. Each of the film clips has been used previously to induce a specific emotional reaction ([e.g., [52]–[54]). See Table 1 for a description of each film clip used to elicit each type of emotion—fear, amusement, disgust, or neutral affect.

**Table 1:** Description and arousal ratings for each emotional film clip.

Name	Brief Description	Length	Emotion	Standardized Arousal Rating (Z-score)
There is Something About Mary*	Ben Stiller fights with dog	2∶55	Happiness	−.01
When Harry Met Sally*	Sally simulates orgasm	2∶53	Happiness	.32
Robin Williams Live at The Met**	Alcohol/Marijuana	5∶59	Happiness	.93
The Shining*	Character pursues wife with Axe	4∶33	Fear	.55
The Shining*	Boy looks for mom	1∶22	Fear	.02
It*	Clown in sewer attracts a boy	1∶56	Fear	.33
Trainspotting*	Character dives into filthy toilet	1∶44	Disgust	.41
Pink Flamingos**	Woman eats dog feces	1∶17	Disgust	1.24
Amputation***	Amputation of arm	1∶08	Disgust	.80
Abstract shapes***	Colors shown across the screen	3.26	Neutral	−1.76
Color bars***	Bars of color shown across the screen	1.31	Neutral	−1.71
Blue (1)*	A piece of foil floating in the air.	1.16	Neutral	−1.13

*Note.* * =  taken from Schaefer, Nils, Sanchez, & Philippot [54]; ** = taken from Rottenberg, Ray, & Gross [53]; *** =  taken from Gross and Levenson [52].

Each of the film-clips has been given a self-report arousal rating, which was published in the article from which the film clip was identified (i.e., [52]–[54]). Film-clips taken from Schaefer et al. [54] were rated on a 7-point Likert Scale ranging from 1 (not at all arousing) to 7 (extremely arousing), whereas film-clips identified in Gross and Levenson [52] and Rottenberg et al. [53] were rated on 9-point Likert Scales ranging from 0 (not at all arousing) to 8 (extremely arousing). To account for the use of different rating scales, we standardized the arousal ratings of the film-clips by converting them into Z-scores. These arousal ratings are listed in Table 1. A one-way ANOVA was conducted to see if the film-clips differed in standardized arousal ratings. *F*(3,9) = 22.20, *p* = .001. This analysis was followed by post hoc LSD tests to determine which specific stimulus types differed in arousal. Amusing film-clips (*M* = .42; *SD* = .48) did not differ significantly in arousal ratings from the scary (*M* = .30; *SD* = .27) or disgusting (*M* = .81; *SD* = .41) film-clips, *M_dif_*  = .11, *SE* = .31, *p* = .725, *M_dif_*  = −.40, *SE* = .31, *p* = .239, respectively. Moreover, the disgusting and scary film-clips did not differ from each other in arousal ratings, *M_dif_*  = .51, *SE* = .31, *p* = .140. However, neutral film-clips (*M* = −1.53; *SD* = .35) were rated as significantly less arousing than amusing, scary, or disgusting film-clips, *M_dif_*  = −1.94, *SE* = .31, *p* = .001, *M_dif_*  = −1.83, *SE* = .31, *p* = .001, *M_dif_*  = −2.35, *SE* = .31, *p* = .001, respectively.

#### Speeches

We used political speeches as a dependent variable because we wanted to assess how the emotional manipulations would affect participant agreement with left and right wing political ideas using an ecologically valid measure. The speeches were found online by conducting Google searches for each topic, and choosing ones written by left- or right- wing politicians who adopted a clear ideological stance on an issue. Participants read four political speeches, written by prominent right- or left-wing politicians, on the following four topics: the war on terror, gay marriage, stem cell research, and immigration. These topics were chosen because they are considered important issues dividing the political left and right [55]. Four speeches per condition were used to ensure the generalizability of the results to political orientation, rather than to narrower identification with a topic-specific opinion. The speeches were not labeled as right- or left-wing, and it was not apparent which politician had written which speech. The speeches were between 800 and 2000 words (*M* = 1744.5; *SD* = 920). The right and left wing speeches did not differ significantly in overall mean length (*t*(3) = 2.15, *p*>.05). Speeches were counter-balanced in their presentation. In a pilot test, 51 participants were asked to indicate “how complex are the ideas outlined in this political speech” on a 9-point likert type response scale ranging from 1 (not at all complex) to 9 (extremely complex). The right-wing (*M* = 4.42, *SD* = 1.86) and left-wing (*M* = 3.96, *SD* = 2.18) did not differ significantly in complexity, *t*(50) = −1.81, *p*>.05, *Cohen's d* = .22.

### Results

Although we recruited an equal number of participants in each experimental condition, because of attrition, the sample sizes were not exactly equal. Between 50–63 participants were ultimately included in each condition (see Table 2).

**Table 2:** Means, Standard Deviation (SD), and number of participants (N) in each group for Study 1.

Condition	Right-wing speeches			Left-wing speeches		
	*M*	*SD*	*N*	*M*	*SD*	*N*
Fear	5.67_b_	1.80	55	6.46_a_	1.80	53
Amusement	5.70_b_	1.87	52	6.39_a_	2.00	51
Disgust	5.12_b_	1.65	50	6.48_a_	1.70	54
Neutral	3.33_a_	2.26	64	6.13_a_	1.95	63

*Note.* Different subscripts within a single column denote significantly different mean values (*p*<.001).

We conducted a univariate analysis of variance (ANOVA) that assessed whether (1) experimental condition (i.e., witnessing an amusing, frightening, disgusting, or neutral film clip), (2) right- versus left-wing content of speech, (3) baseline political orientation, and interactions among these factors, would predict agreement with political speeches. Partial eta-squared effect sizes were examined for all ANOVA analyses. This statistic is equal to the percent of variation in the dependent variable that is accounted for by the independent variable(s).

#### Main effects

We first examined the basic main effects of baseline political orientation, speech type, and film type upon agreement with political speeches. An overall main effect of political orientation upon agreement with political speeches was not found, *F*(1,441) = .17, *p* = .685, *η^2^*<.01. On the other hand, a main effect for speech type emerged, *F*(1,441) = 251.37, *p* = .001, *η^2^* = .37, such that participants tended to agree more, overall, with left-wing (*M* = 6.35; *SE* = .13) compared to right-wing (*M* = 4.97; *SE* = .13) speeches. The finding that participants preferred left-wing speeches is consistent with previous findings that participants recruited through MT tend to be more left-leaning (e.g., [47]).

A main effect of film type on agreement with political speeches also emerged (*F*(1,439) = 5.06, *p* = .002, *η^2^* = .03). LSD post-hoc tests revealed that the three different types of emotion-inducing films each elicited significantly greater endorsement with political speeches compared to neutral film-clips, *M_diff_*  = −.81; *SE* = .21; *p* = .001; *M_diff_*  = −1.02; *SE* = .21; *p* = .001; *M_diff_*  = −1.02; *SE* = .21; *p* = .001, for disgusting, scary, and amusing, respectively. The three different types of emotion-inducing film-clips did not differ in their capacity to alter agreement with political speeches (*ps*>.05). Emotional arousal therefore led participants to agree more with speeches in general. This finding appears consistent with research that has suggested that arousal tends to enhance liking of stimuli (e.g., [56]–[57]).

#### Interactions

Not surprisingly, an interaction emerged between baseline political orientation and left- versus right-wing content in predicting agreement with political speeches, *F*(1,441) = 176.92, *p* = .001, *η^2^* = .29, whereby participants with more conservative versus liberal orientation preferred right- versus left-wing speeches. Political orientation and experimental condition did not interact to predict agreement with political speeches, *F*(1,439) = .72, *p* = .542, *η^2^* = .01.

**Figure 1:**
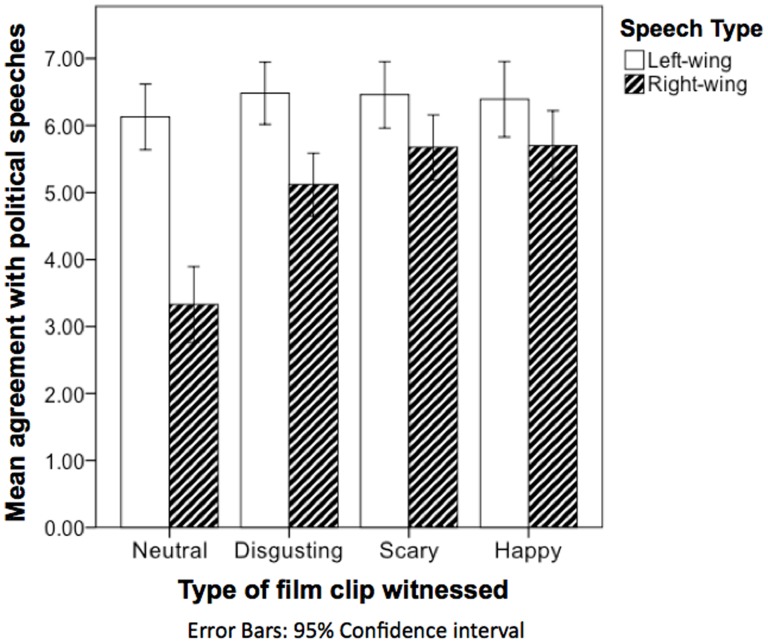
Mean agreement with political speeches among individuals who have just witnessed an arousing or neutral film clip.

Our main hypothesis specified that watching an arousing film clip would lead to enhanced agreement with right-wing and reduced agreement with left-wing political speeches compared to watching a neutral film-clip, while controlling for the effect of baseline political orientation. Evidence for the validity of this hypothesis would be provided by the existence of a significant interaction between the effect of film type and left- versus right-wing content upon agreement with political speeches. Such an interaction indeed emerged, *F*(1,439) = 5.25, *p* = .001, *η^2^* = .04. Follow up post hoc LSD tests suggested that, as hypothesized, each type of emotional film clip was associated with enhanced agreement with right-wing speeches compared to neutral film clips (*M_diff_*  = −1.33; *SE* = .31; *p* = .001; *M_diff_*  = −1.71; *SE* = .30; *p* = .001; *M_diff_*  = −1.78; *SE* = .21; *p* = .001, for disgusting, scary, and amusing, respectively; See Table 2; Figure 1). None of the emotional stimulus types differed significantly in terms of their ability to heighten agreement with right-wing speeches (*p*s>.05). Unexpectedly, different types of film-clips did not elicit significantly altered magnitudes of agreement with left-wing speeches (*p*s>.05) (see Table 2; Figure 1). This implies that arousal may enhance agreement with right-wing content, without necessarily affecting endorsement of left-wing content.

A 3-way interaction between baseline political orientation, type of film-clip witnessed, and right- versus left-wing content, was not found to predict agreement with political speeches, *F*(1,439) = 1.82, *p* = .143, *η^2^* = .01. This suggests that the experimental manipulation did not differentially affect liberals and conservatives.

We re-ran our analyses, this time analyzing the specific arousal ratings listed in Table 1 rather than the four different valence categories of film-clips (i.e., neutral, fear, amusement, disgust). A main effect of arousal upon agreement with political speeches was noted, *F*(1,441) = 2.05, *p* = .024, *η^2^* = .07, once again suggesting that arousal may enhance overall agreement with political speeches. Arousal did not interact with baseline political orientation to predict overall agreement with political speeches, *F*(1,441) = 1.09, *p* = .313, *η^2^* = .19. However, in support of our main hypothesis, a significant interaction was noted between arousal and endorsement of right versus left wing content speeches, *F*(1,441) = 4.76, *p* = .001, *η^2^* = .14, whereby arousal was associated with enhanced agreement with right-wing (*F*(1,441) = 6.68, *p* = .001, *η^2^* = .31) but not with left-wing (*F*(1,441) = .76, *p* = .679, *η^2^* = .06) speeches. This analysis provides further evidence that arousal modulates agreement with right-wing speeches, specifically.

A 3-way interaction was once again not found between baseline political orientation, experimental condition, and right versus left-wing content, *F*(1,441) = 1.17, *p* = .222, *η^2^* = .15. This provides further evidence that the experimental manipulation did not differentially impact liberals and conservatives.

### Study 2

Study 1 suggested that political conservatism is motivated by positively as well as negatively-valenced emotional arousal and thus more broadly than previously considered. Study 2 similarly induced positive and negative emotional arousal states to see if this would lead to conservative shifts in beliefs, controlling for baseline political affiliation. Additionally, Study 2 assessed whether the amplification of such positive and negative emotional arousal would further modulate agreement with right-wing political speeches.

In Study 2, participants were again asked to view a positive, negative, or neutral film-clip, and then to read–and rate their agreement with–political speeches. This time, participants who were assigned to view an emotionally arousing film-clip were either asked to amplify their emotional reactions to the emotional film-clip or to watch the emotional film-clip naturally. We hypothesized that instructions to amplify emotional reactions to an emotion-inducing film-clip would intensify the degree to which arousal leads to the endorsement of conservative political beliefs. Instructions to amplify emotional reactions to positive and negative film-clip-stimuli has been found to enhance the emotional experience, emotion-expressive behavior, and autonomic physiology, associated with the affective content of film-clips compared to when participants are instructed to watch such film-clip-stimuli naturally [58]. Therefore, if emotional arousal leads to the endorsement of conservative political ideology, then participants instructed to amplify their emotional reactions to positive and negative emotion-inducing film-clips should endorse more conservative political beliefs compared to those instructed to watch such film-clips naturally.

With this study, we sought to assess (1) whether we would replicate the finding that watching an arousing film-clip—whether positive or negative–would elicit greater agreement with right-wing speeches compared to watching a neutral film-clip and (2) whether this effect would be enhanced by asking participants to amplify their emotional reactions to the emotional film-clip. Only right-wing content speeches were employed in Study 2, because arousal was not found to modulate agreement with left-wing political speeches in Study 1.

### Method

#### Participants

350 American participants were recruited through MT and were compensated $1.40 for their time. Due to attrition, complete data was obtained for 277 participants (129 males; mean age  = 31.23 years; *SD* = 11.50; range  = 18–70).

#### Political orientation

Baseline political orientation was assessed with the same item employed in Study 1. The sample mean was 3.67 (*SD* = 1.70).

#### Procedure

The research protocol was approved by the University of Toronto ethics board. All participants gave their consent before beginning the online survey.

Study 2, like Study 1, employed a between-groups design. Participants were randomly assigned to watch either an emotion-inducing (amusing or scary) or a neutral film clip and then to read right-wing content speeches.

The right-wing content speeches were the same ones used in Study 1 and participants rated their level of agreement with these speeches using the same 9-point Likert scale employed in Study 1. The amusing, scary, and neutral film clips were the same ones employed in Study 1. This time, we did not administer any disgust-inducing emotional film-clips. This was because such film-clips did not yield significantly different magnitudes of conservative shifts in political beliefs in Study 1 compared to fear or amusement inducing film-clips and we therefore did not have any hypotheses about specific negative emotional states. As in Study 1, we used three different film clips of each type to ensure that the effects were not stimulus specific.

Participants who were assigned to watch a neutral film clip received no instructions. Those who were assigned to watch an emotional film-clip were randomly assigned to either amplify their reactions to that film-clip or to watch the film-clip naturally. The instructions, which are modified from Gross & Levenson [59], are as follows:

*Natural film-clip*: You are about to be directed to watch an anxiety-provoking [pleasant] video clip. It is quite normal that viewing such a clip will create some level of discomfort or fear [amusement]. Please try to experience your feelings without trying to control or change them in any way. Please let your feelings run their natural course.

*Amplify film-clip*: You are about to be directed to watch an anxiety-provoking [pleasant] video clip. It is quite normal that viewing such a clip will create some level of discomfort or fear [amusement]. Please try to amplify your feelings as you view the film clip. Please behave in such a way that a person watching you would know you were feeling scared [amused].

We opted to modify the paradigm employed by Gross & Levenson [59] as little as possible because it has previously been found to successfully manipulate emotional intensity. For this reason, participants were warned about the type of film-clip that they were about to watch (amusing or anxiety-provoking) in replication of Gross & Levenson [59], even though this may have caused some priming effects.

### Results

Although we recruited an equal number of participants in each experimental condition, not all participants completed the study and so the sample sizes were not exactly equal. Between 42–70 participants were ultimately included in each condition (see Table 3).

**Table 3:** Means, Standard Deviation (SD), and number of participants (N) in each group for Study 2.

Condition	Right-wing speeches		
	*M*	*SD*	*N*
Fear (amplify reaction)	5.85_c_	1.25	42
Fear (watch naturally)	4.37_b_	2.10	50
Amusement (amplify reaction)	5.70_c_	1.47	70
Amusement (watch naturally)	5.18_c_	1.58	49
Neutral	3.12_a_	2.28	66

*Note.* Different subscripts within a single column denote significantly different mean values (*p*<.01).

We conducted two ANOVAs that assessed (1) whether watching a positive, negative, or neutral film-clip would predict agreement with right-wing speeches while controlling for the effect of baseline political orientation, and (2) among participants who watched an emotional film-clip, whether valence (positive vs. negative), instructional manipulation (amplify vs. watch naturally), and an interaction between these two factors, would predict agreement with right-wing speeches while controlling for the effect of baseline political orientation. Partial eta-squared effect sizes were examined for all ANOVA analyses.

In our first analysis, experimental condition (positive vs. negative vs. neutral) and baseline political orientation were entered into the model as factors. We assessed whether these two variables, and/or an interaction between them, would predict mean agreement with the right-wing political speeches. Not surprisingly, a main effect was noted whereby baseline political orientation predicted agreement with right-wing speeches, *F*(1,277) = 19.30, *p* = .001, *η^2^* = .14. Moreover, a main effect of experimental condition was noted, *F*(1,275) = 3.55, *p* = .032, *η^2^* = .06. Follow-up post hoc LSD tests revealed that, in replication of Study 1, watching an arousing film-clip–whether positive or negative–elicited significantly greater agreement with right-wing speeches compared with watching a neutral film-clip, controlling for baseline political orientation, *M_diff_*  = −2.28; *SE* = .36; *p* = .001; *M_diff_*  = −2.58; *SE* = .35; *p* = .001, for negative and positive, respectively). Also in replication of Study 1, positive and negative film clips did not elicit significantly different magnitudes of agreement with right-wing speeches, *M_diff_*  = −.30; *SE* = .33; *p* = .367. Finally, in replication of Study 1, baseline political orientation did not interact with the experimental manipulation to predict agreement with right-wing speeches, *F*(1,275) = .43, *p* = .650, *η^2^* = .01.

In our second analysis, valence (positive vs. negative), instructional manipulation (amplify vs. accept), and baseline political orientation were included in the model as factors. We assessed whether these variables, and/or interactions among them would predict mean agreement with right-wing political speeches.

**Figure 2:**
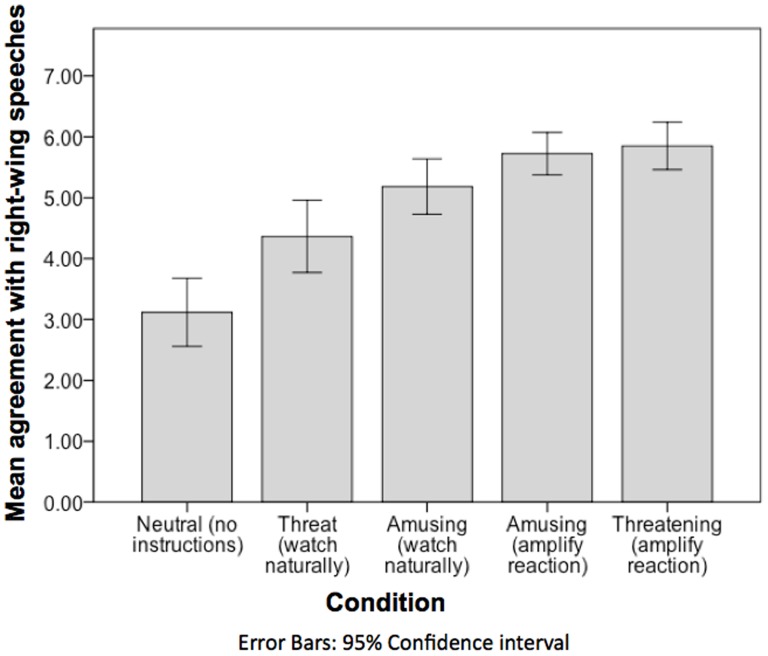
Mean agreement with right-wing political speeches among individuals who have just witnessed a neutral film clip or an arousing film-clip with instructions to either watch naturally or to amplify emotional reactions.

A main effect was noted whereby baseline political orientation predicted agreement with right-wing speeches, *F*(1,277) = 24.61, *p* = .001, *η^2^* = .11. A trend of main effect was also noted whereby positively valenced film clips may have elicited more agreement with right-wing speeches than negatively valenced film clips, *F*(1,277) = 3.50, *p* = .063, *η^2^* = .02. See Figure 2 and Table 3 for means and SDs.

In support of our study hypothesis, a main effect of instructional manipulation was also noted whereby instructions to amplify reactions to emotional film-clips led to more agreement with right-wing content compared to watching such film-clips naturally, *F*(4,275) = 21.36, *p* = .001, *η^2^* = .10. Unexpectedly, we also noted a significant interaction between valence and instructional manipulation, *F*(1,275) = 9.34, *p* = .003, *η^2^* = .04. Post hoc LSD tests revealed that participants who were instructed to enhance their emotional reactions to the negative film-clips agreed more with the right-wing film-clips than participants who were instructed to watch the negative film clips naturally, *M_diff_*  = −1.66; *SE* = .33; *p* = .001. On the other hand, participants who watched positive film-clips agreed with the right-wing speeches to the same extent regardless of whether they did or did not amplify their emotional responses, *M_diff_*  = −.31; *SE* = .29; *p* = .290. (See Figure 2 and Table 3 for mean levels of agreement with right-wing political speeches for each condition). Thus, although amplifying emotional arousal tended to elicit more agreement with right-wing speeches, this effect seemed limited to negatively-valenced clips. We did not anticipate this, but wonder if it was caused by a ceiling effect. Participants who watched amusing film clips seemed to agree with the right-wing speeches at a rather high level, even when they watched the film naturally.

Finally, we noted that baseline political orientation did not interact with the amplify/watch naturally experimental manipulation, *F*(1,275) = 1.98, *p* = .161, *η^2^* = .01, nor was there a 3-way interaction between baseline political orientation, instructional manipulation, and valence of film-clip watched, *F*(1,275) = .12, *p* = .843, *η^2^*<.01. Once again, this suggests that our experimental manipulation did not differentially affect liberals and conservatives.

## General Discussion

In a series of two studies, participants watched film clips previously shown to elicit positive, negative, or neutral affect in order to determine their impact upon agreement with multiple speeches of left- and right-wing ideological content. Both positive and negative stimuli induced enhanced agreement with right- but not left-wing political speeches compared to neutral stimuli. These findings may suggest that conservative political orientation is motivated more broadly than previously considered given the emphasis current theories place upon the relationship between ideology and specifically aversive states such as threat and disgust (e.g., [3], [7]–[9], [12]; see [2], [4] for reviews).

Our study does not support a negative valence model of political ideology, and furthermore, does not support extremism theories of political beliefs (e.g., see [25], [60]). According to such theories, threat should motivate extreme political beliefs, regardless of orientation, such that liberals become more extreme in their liberalism and conservatives more entrenched in their conservatism. We did not find this type of interaction between baseline political beliefs and experimental induction of positive, negative, or neutral affect. Rather, an overall effect was noted in which emotional arousal enhanced agreement with specifically right-wing political content.

The finding that positive emotion may enhance conservatism suggests a potential causal mechanism to explain the previously noted correlations between happiness and conservatism (e.g., [61]–[64]). Although researchers have assumed that conservatism enhances psychological well-being (e.g., [62]–[63]), our research suggests that happiness may, as well, enhance conservative political orientation.

More broadly, our study suggests that arousal rather than negative valence may underlie the conservative shifts in political beliefs that have been found to follow from experimental induction of threat (e.g., [6]–[9]) and disgust (e.g., [10]–[13]). Threat is argued to be one of the most highly arousing emotions (e.g., [16]–[18]). Disgust, as well, is thought to be a highly arousing emotional state and there is an important evolutionary basis for this (see [65]). Thus, researchers may have mistakenly assumed that conservative shifts were caused by negative valence rather than by arousal.

Two recent electrophysiological studies support the role of arousal rather than valence as the affective component of politically conservative orientation [23]–[24]. These studies suggested that arousal rather than negativity underlies emotion-processing biases among conservatives. However, while these studies illuminate trait-level individual differences in arousal sensitivity among conservatives, the present study uniquely presents *causal* evidence that both positive and negative emotional states prompt state-level changes in the endorsement of conservative belief. Taken together, it seems that conservatives respond to emotional stimuli with enhanced intensity, and that such emotional arousal may further promote their conservative orientation.

Future studies are needed to investigate the mechanism through which arousal may lead to conservative shifts in beliefs. Two possibilities are: (1) that arousal motivates individuals to endorse value systems that promote a world in which the potential for intense arousal is minimized, and (2) that arousal interferes with cognitive ability, which causes a preference for intuitive and conservative ideas.

### Additional directions for future research

Although the studies described and reviewed here may broaden our understanding of the psychological underpinnings of political belief, several important unanswered questions remain:

1. The topics of the political speeches used in the present studies relate exclusively to social issues. Future research is needed to see if our findings generalize to economic political issues. This is important because social and economic conservatism may be distinct ideologies that appeal to different groups of individuals. For instance, social conservatism has been found to be most prevalent among conservative Protestants, while economic conservatism is most popular among individuals in high-income brackets. Furthermore, there is some evidence that social conservatives are less open-minded and more dogmatic than economic conservatives [66].
2. The current study did not reveal any affect of arousal upon endorsement of liberal speeches, and it remains unclear why. If political orientation is best represented along a single dimension ranging from liberalism to conservatism, then we would expect that if arousal enhances conservative political beliefs, it should also reduce liberal political beliefs. Alternatively, however, arousal may affect participant liking of conservative speeches, without altering their feelings towards liberal speeches. Theoretically, this explanation may be valid if political orientation is not best represented by a single dimension ranging from liberalism to conservatism, as described previously.
3. We did not find an interaction between baseline political orientation and emotion-induction in promoting enhanced agreement with right-wing content political speeches. If conservatives are intolerant of arousal and that is implicated in the mechanism through which arousal enhances agreement with conservative speeches, then we would expect the effect of arousal upon endorsement of right-wing political speeches to be greatest among conservatives. Our limited sample size and restricted range of political orientation (more liberal than conservative) may have hindered our power to find this. Future studies with more diverse samples are needed to investigate whether baseline political orientation interacts with emotional-inductions to promote the endorsement of conservative political ideas. Moreover, we employed a 1-item measure of baseline political orientation. Future studies might beneficially employ more comprehensive measures of political attitudes as baseline and dependent variable assessments. This might allow for more power to detect effects of experimental manipulations and would enhance confidence in the findings.
4. The film-clips that we employed have been documented to elicit only one specific type of positive mood–amusement [52]–[54]. Future studies should assess whether other positive mood states enhance conservative ideology. For instance, if intense emotional arousal leads to conservative shifts in beliefs, then arousing positive-mood states such as excitement should enhance endorsement of right-wing ideology but calming positive states such as contentment should not. Such replication with a wider variety of positive-mood inducing stimuli will furthermore help to establish the reliability/validity of our findings. Additionally, and more generally, stimuli that differ in motivational salience tend to be experienced asymmetrically in terms of positive or negative valence, and this may need to be accounted for in future research. For instance, according to *positivity offset* theory, individuals tend to respond more positively to stimuli that are relatively low in arousal, whereas *negativity bias* theory suggests that individuals may react more negatively to stimuli that are relatively high in arousal (e.g., [67]). Future studies should accordingly systematically experimentally induce positive and negative mood states varying in arousal levels.
5. Finally, it remains unclear why amplification of positive material did not modulate endorsement of right-wing speeches. This may have been due to a ceiling effect in which participants that watched amusing film clips agreed with the right-wing speeches at a rather high level, even when they watched the film naturally. Research is needed to replicate and to understand this effect given that it was unanticipated.

## Conclusion

Our study suggests preliminary support for a generalized arousal model of political belief, which may reflect more precisely the psychological motivations underlying conservatism. Developing an accurate and all encompassing theoretical model of the psychological basis of political orientation will facilitate a better understanding of the self-regulatory psychological functions served by political beliefs.
